# Supplementary material for: Are carbon emissions trading and green financial instruments synergistic? -Comprehensive quantitative research based on content analysis
Source: PLoS One. 2024 Mar 7;19(3):e0298601. doi: 10.1371/journal.pone.0298601 (PMC10919601; doi:10.1371/journal.pone.0298601)
Supplement: S1 Data — (ZIP) [file pone.0298601.s001.zip › Data/地级市Z_F工作报告.zip!/╣π╢1⁄2/╜╥╤⌠╩╨/└┤╫╘╣1⁄2╓┌║┼í╛┬φ┐╦╩2╛▌═°í┐_2005.docx]

各位代表：

我代表市人民政府向大会作政府工作报告，请予审议，并请政协各位委员和其他列席人员提出意见。

2004年工作回顾

2004年，在省委省政府和市委的正确领导下，在市人民代表大会及其常务委员会和市政协的监督支持下，市政府坚持以“三个代表”重要思想和科学发展观为指导，认真贯彻落实中共十六届三中、四中全会，省委九届四次、五次全会和张德江书记考察粤东重要讲话精神，紧紧围绕年初确定的奋斗目标，以抢抓机遇、加快发展为主题，以工业发展为龙头，以推进重点项目建设为突破口，发展壮大县域经济，全市经济社会继续保持平稳发展。全年生产总值540.53亿元，比上年（下同）增长7.2%。

一、成功抗击疫情旱情，农业农村经济稳步发展

在年初发生高致病性禽流感疫情的情况下，采取坚决有力措施，依靠科学、依靠法制、依靠群众，全力以赴抗击疫情，取得了抗击高致病性禽流感疫情的重大胜利，受到国家有关部门和省的充分肯定。同时，战胜了冬春连旱灾情，农业农村经济稳步发展，农林牧渔业总产值56.17亿元，增长3.9%。规模化生产不断扩大。粮食生产呈现恢复性增长。6大农业支柱产业、12大农业商品基地建设水平不断提高。新种水果5.3万亩、茶叶0.2万亩、竹笋1.2万亩，水产品总产量14.13万吨。揭东、揭西分别建成全省最大野鸭和全国最大鹧鸪生产基地，普宁、惠来分别进入全国水果、水产百强县行列，惠来被评为“中国荔枝之乡”。标准化建设和推广不断增强。普宁果梅、蕉柑、揭东竹笋、柚木、揭西青榄5个国家级和14个省、市级农业标准化示范区建设顺利推进。新制定7个农业地方标准，累计23个。无公害、绿色、有机农产品基地建设取得突破性进展。产业化水平不断提高。15家省、市级农业龙头企业和27家年销售额1000万元以上农产品加工企业发展势头良好，揭西京明茶叶公司被省评为优秀扶贫农业龙头企业，85个农产品市场设施不断配套完善，基本形成生产有基地、加工有龙头、销售有市场的产业化格局。城镇化进程不断推进。16个中心镇新一轮总体规划修编步伐加快，其中5个已完成规划纲要评审并进入成果报批。扶贫开发力度不断加大。投入扶贫资金1.45亿元，扶持“三高”农业、基础设施建设等项目520个。404名农村贫困家庭子女免费就读技工学校，138名贫困生就读“勤工俭学”扶贫班。农村税费改革继续深化，农民负担进一步减轻。

二、努力发展第二产业，工业经济实力逐步增强

把工业作为经济发展的龙头来抓，加快优势产业集聚，工业生产保持较快增长。工业总产值1061.24亿元，增长8.5%。工业对经济增长的贡献率达56.4%。工业用电量29.32亿千瓦时，增长29.7%。“三个新增100”取得成效。完成1000万元以上投资额的项目87个，投入资金34.07亿元；完成500万元以上投资额的工业项目113个，投入资金29.26亿元；新增规模以上企业110家，累计564家，规模以上工业产值252.64亿元，增长18.1%。“668”计划稳步推进。6城工业产值11.52亿元，增长16.7%；6基地工业产值395.21亿元，增长10.2%；8园区工业产值75.45亿元，增长12.3%。民营工业增速加快。全年产值869.6亿元，增长7%，占工业总产值的81.9%。技术创新能力不断增强。榕泰AV抗菌复合材料等13个项目列入省重点技改和创新项目计划，全年完成技改项目38项。巨轮、运通公司成功上市。新增省名牌产品5个、著名商标14件，累计分别为13个、34件。“顺达”牌不锈钢压力锅获中国名牌产品称号，实现我市国家级名牌产品零的突破。

三、有效促进经贸工作，市场流通态势稳中趋旺

积极开展多种形式的经贸活动，大力开拓国内外市场。对外经贸有新的发展。新增外贸经营企业86家，累计303家。外贸出口9.5亿美元，增长38.6%。新签利用外资合同26宗，合同利用外资1.32亿美元，增长4.9%；实际利用外资0.84亿美元，下降41%。会展经济有效拓展。成功举办第四届普宁（国际）衬衣节暨纺织服装交易会、第三届中华阳美（国际）玉器节和揭货（成都）展销会，参加省组织的“珠洽会”、“山洽会”、中小企业博览会、服装博览会等经贸活动，共签约33.5亿元。消费热点逐步形成。旅游、住房、汽车、通讯、餐饮娱乐、医疗保健等消费市场和服务业稳步发展。全市社会消费品零售总额185.24亿元，增长11.2%。

四、切实抓好重点项目，基础设施建设不断加强

在国家继续实施宏观调控政策的情况下，积极想办法，多元化筹集资金，加快重点项目建设。全社会固定资产投资86.43亿元，增长1.1%。重点工程建设步伐加快。市区临江南路综合工程拆迁和建设进展顺利，堤围和窖园建设将于近期竣工。城乡水利防灾减灾工程建设步伐加快，在建10宗工程累计投资2.86亿元。惠来石碑山风电场已投入1亿多元，正在做好变电站、场内道路和风机基础等各项工作。500千伏榕江输变电工程、汕揭和潮揭高速公路揭阳段等一批项目正在加快建设。市区垃圾处理场、莲花大道南段、4条样板路等市政重点工程正在抓紧推进。第二期农网改造工程国债项目通过省竣工验收。项目申报核准进展顺利。惠来电厂已完成地表清理，正在做好相关基础工作，上报国家发改委核准。潮汕民用机场已确定投资主体，成立领导机构，正在抓紧编制可行性研究报告。

五、逐步规范财税秩序，财政金融运作保持平稳

强化税收征管，积极组织收入，依法治税理财，稳妥化解财政风险和金融风险，财政金融正常运作。财政运行质量逐步改善。剔除不合理非税收入2.01亿元后，地方财政一般预算收入9.64亿元，下降8.93%，其中工商税收6.04亿元，增长20.15%，成为五年来最高增幅。非税收入占财政一般预算收入的比重由上年的44.87%下降为28.42%，收入结构渐趋优化，为今后高速可持续增长奠定了基础。地方财政一般预算支出29.27亿元，下降5.44%。重点支出保障力度进一步加大。金融保持稳步发展。年末全市金融机构各项存款余额407.18亿元，贷款余额171.17亿元，分别比年初增长18.4%、3.1%。

六、注重抓好协调发展，社会各项事业全面进步

在加强经济建设的同时，努力促进社会各项事业协调进步。教育事业稳步发展。“普九”成果得到巩固和提高。社会力量办学有新进展。中小学布局调整力度加大。完成第二批100所老区山区破旧小学改造工作，第三批改造工作正在全面铺开。揭阳一中、市技校新校区建设进展顺利。科技工作得到加强。新设立1个省级工程技术研究开发中心，累计4个。新增2家国家级、5家省级高新技术企业和11家省级民营科技企业，总数分别为8家、30家和101家。列入国家和省各类科技计划56项，通过省、市级科技成果鉴定33项。人才工作得到重视，引进力度进一步加大。文化事业扎实推进。举办揭阳市首届戏剧文化节和“书香节”、揭阳先贤史定（揭猛）学术研讨会等主题活动，榕城区文化馆、普宁市文化馆被评为国家一级馆，普宁被省评为“篮球之乡”，泥沟村被评为“广东省民族民间艺术之乡”。音像复制业和印刷出版业等文化产业进一步发展。卫生事业发展迅速。建立市卫生应急指挥中心，公共卫生体系建设逐步健全。疾病控制、妇幼保健工作取得成效，卫生监督力度加大。新型农村合作医疗工作有新的发展，全市参加新一年农村合作医疗215万人。民心工程进一步推进。认真落实就业和再就业政策，加快劳动力市场建设，成功举办城乡统筹就业招聘大会，新增就业岗位2万个，下岗失业人员实现再就业1.07万人，城镇登记失业率控制在2.7%以内。“两个确保”和“三条保障线”得到较好落实，城乡保障体系进一步完善。积极帮扶弱势群体，切实做好赈灾救济工作，支出低保资金3698万元，保障了3.6万户10.3万困难群众基本生活。解决了78万群众饮水难问题，群众“四难”问题得到有效缓解。城乡居民生活水平不断提高，农民人均纯收入3949元，增长3%；市区城镇居民人均可支配收入8481元，增长9%。其他各项事业全面进步。拥军优属、民兵预备役和征兵工作取得新的进展。审计、统计工作得到加强，经济普查工作有序开展。体育、气象、地震、外事侨务、民族宗教、人民防空、广播电视、文史档案等各项事业得到发展。

七、不断加强各项管理，社会局面基本保持稳定

切实把加强社会管理、保持社会稳定作为坚持执政为民、构建和谐社会的大事抓紧抓好。市场秩序进一步规范。制假售假、走私贩私、偷税逃税等违法犯罪活动受到有力打击，食品、药品整治得到加强，清理无照经营1.2万户，市场环境逐步优化。妥善处置“4·8”仙桥触电事故等突发事件，深入开展交通运输、企业安全生产、危险化学品等专项整治，安全生产形势基本稳定。生态保护进一步加强。计生工作朝着依法有序方向发展，全年人口出生率12.35‰，自然增长率7.49‰，计划生育率87.4%，通过了省考核。殡葬改革成果得到巩固，火化率保持100%。森林、土地等资源保护力度加大，生态公益林体系、绿色通道及三年灭荒工程有效实施，森林覆盖率51.5%。环境保护工作得到重视，出台“三江”水源保护措施，工业污染源治理工作逐步落实。治安形势进一步好转。严厉打击各种严重刑事犯罪活动，深入开展打黑除恶以及打击“两抢两盗”、“六合彩”赌博等专项整治斗争，切实推进“禁毒03工程”，普宁摘除“毒品重灾区”帽子。基层调解、信访工作得到加强，成功处置解决非正常集体上访等群体性突发事件，社会保持稳定。

八、重视塑造政府形象，民主法制建设扎实推进

坚持科学行政、民主行政、依法行政，切实转变政府职能，加强作风建设，塑造政府良好形象。民主建设步伐加快。自觉接受法律监督和民主监督，重大问题认真征求人大代表、政协委员意见，加强与各民主党派、工商联和工青妇等群团组织的联系，广泛听取人民群众对政府工作的意见和建议，民主决策机制进一步完善。认真办理人大代表议案、建议和政协提案，共办理人大代表建议61件、政协提案87件。完善村（居）民自治和企事业单位民主管理制度，推行政务、村务、厂务公开，进一步扩大基层民主。依法行政水平不断提高。认真贯彻实施行政许可法，制定市政府规范性文件6件，废止涉及行政许可规定的政府规范性文件26件，取消行政许可项目88个。继续深化行政管理体制改革和行政审批制度改革，强化政府社会管理和公共服务职能。作风建设深入开展。郑重做出并严格执行五项廉政承诺，进一步落实廉政建设责任制，严肃查处违法违纪案件。加强公务员队伍建设，改进机关作风，提高行政效能和办事效率。

各位代表！过去一年，我市经济建设和社会事业在比较困难的情况下，仍然保持平稳发展势头。这是省委省政府和中共揭阳市委正确领导的结果，是各级人民代表大会及其常务委员会和人民政协监督支持的结果，是全市人民团结奋斗、攻坚克难、开拓进取的结果。我代表市人民政府向全市人民，向中央和省驻揭单位、驻揭人民解放军、武警官兵表示崇高的敬意！向长期关心支持揭阳发展的港澳台同胞、海外侨胞、海内外乡亲和友好人士表示衷心的感谢！

回顾过去一年的工作，我们清醒地看到，我市经济社会还存在不少困难和问题。主要表现在：整体经济素质不高，产业竞争力不强，发展速度偏慢；投资环境不如人意，招商引资效果不佳；固定资产投资拉动乏力，市区建设管理滞后，城镇化进程不快；县镇村债务包袱沉重，地方财力支撑脆弱；社会事业欠账较多，基础教育发展缓慢，环境污染日趋严重；“三农”问题仍较突出，部分群众生活还较困难；群体性事件隐患仍然存在，社会稳定压力加大，社会管理有待进一步规范；政府职能转变相对滞慢，公务员队伍素质亟须迅速提高。对这些问题和困难，我们要高度重视，采取措施，标本兼治，在加快发展中逐步解决。

2005年工作安排

今年市政府工作的指导思想是：以邓小平理论和“三个代表”重要思想为指导，树立和落实科学发展观，认真贯彻中共十六届四中全会、胡锦涛总书记视察广东重要讲话和省委九届五次、六次全会以及市委三届三次全会精神，围绕建设“工业强市、文化大市、和谐揭阳”的战略目标，按照组织工业发展、城镇建设、基础教育“三个新突破”，打造沿江、中部、沿海、西部“四大产业带”，实施能源、交通、水利、环保、文化“五大工程”的发展思路，迅速推进经济发展，加快重点项目和城市建设，全面发展社会事业，推进民主法制进程，强化社会管理，努力实现经济社会全面协调可持续发展。

今年我市经济社会发展主要预期目标是：生产总值增长11%，工业总产值增长14%，农林牧渔业总产值增长4%，固定资产投资增长20%，地方财政一般预算收入增长19.2%，外贸出口增长18%，实际利用外资增长20%，社会消费品零售总额增长13%，居民消费价格总水平上涨控制在3.5%左右，城镇登记失业率控制在3.5%以内，人口自然增长率控制在7.8‰以下。

实现今年预期目标，必须推进十方面工作：

一、推进“工业强市”，壮大经济总量

做大做强支柱产业。坚持走新型工业化道路，调整优化工业产业和产品结构，按照轻重并举、大小结合、新老共进的发展思路，大力发展集约型、循环型产业。继续实施“668”计划，发展壮大五金机械、纺织服装、医药化工、食品饮料、电子信息等支柱产业。狠抓技术改造和创新，大力发展高新技术产业，抓好企业信息化工程，以信息化带动工业化，培育发展新的支柱产业。依托龙头企业，组建行业协会，构建产业公共服务平台，发挥技术研发、质量检测、教育培训、信息交流和物流配送等功能，提高产业集群发展水平。

培植壮大骨干企业。落实领导干部挂钩扶持重点企业制度。继续实施“三个新增100”，在用电补贴、贷款贴息、专项资金等方面扶持优势企业，推动一批企业做大做强。引导骨干企业推进科技、体制和管理创新，鼓励龙头企业加快股份制改造，并争取成为上市公司。今年要在培育大企业和企业集团上有新的突破。力争通过4年的努力，使年销售收入5亿元以上10亿元以下的企业有15家，10亿元以上20亿元以下的有10家，20亿元以上的有4家；年纳税额5000万至1亿元的企业有11家，超亿元的有4家。

积极打造品牌产品。大力实施名牌带动战略，落实奖励措施，扶持发展现有品牌，积极鼓励有条件的企业申报知名商标、著名商标、驰名商标以及国家、省名牌产品和国家免检产品，培植一批带动能力强、拥有自主知识产权的企业和品牌。力争今年实现驰名商标零的突破。

整合提升工业园区。创新办园模式，坚持企业为主、政府推动、市场运作、滚动发展，适当将工业园区管理权和经营权分开，改变园区管理行政化的做法。整合优化园区资源，提升园区的档次和水平，打造园区品牌。重点抓好揭阳试验区、揭东试验区和惠来沿海试验区等工业园区的建设和管理。协助做好汕揭沿海石化产业带的建设规划，积极争取在惠来设立石化工业区。

千方百计招商引资。成立专门领导机构和工作机构，加强协调领导，对招商引资工作一路开绿灯。根据各地吸收外资的基础和发展水平，将全市9个县（市、区）分为4类地区，下达4年吸收外资工作任务。实行招商工作责任主体下移，落实相应奖罚措施，调动各级招商引资积极性和主动性。推进观念更新和体制创新，构建专业化、市场化招商网络，提高招商引资实效。开展涉及企业收费专项整治，努力降低企业营商成本，尽快设立市行政服务中心，不断改善中介服务、技术服务、培训服务，进一步优化投资软环境。积极引进大集团、大公司特别是世界500强企业到我市落户或设立生产、研发基地。做好集聚民心民资民力工作，争取揭籍在外经济大户回乡投资置业。

二、推进“三农”工作，发展农村经济

调整优化种养结构。全面落实种粮补贴等政策措施，稳定130万亩基本粮田面积，确保完成粮食生产任务。巩固提高6大农业支柱产业、12大农业商品基地发展水平，着重建设好全国最大蕉柑生产基地。大力发展效益农业，着力培育特色农业，重点发展特禽、草食动物，积极发展现代渔业，综合开发海洋经济。推进农业标准化生产，加快发展无公害、绿色、有机农产品，实施“绿色证书”工程，全面提高种养业的生产经营和管理水平。 提高产业化水平。发展壮大18家省、市级农业龙头企业，加大3家农业龙头企业培育对象的发展力度，以企业促基地带农户。建立健全科技创新推广和服务体系，发展农产品储藏、运输和加工业，提高农产品附加值。培育农村市场体系，新建和完善蕉柑、青梅、竹笋、荔枝等农产品专业市场，形成一批辐射面广、吞吐量大的现代化农产品物流中心。加强农业信息网络服务，发展订单农业和农产品网上交易，建立鲜活农产品运输“绿色通道”，提高农业经济效益。

加快城镇化进程。高质量修编市域、县域城镇体系规划，抓紧完善包括县城在内的16个中心镇规划。加强城镇基础设施、公共服务设施建设，逐步完善城镇功能配套，促使工业进园、住宅进区、农民进厂，增强城镇辐射和带动农村的能力。各县（市、区）要加大城区建设力度，确保每年筹集1亿元以上资金投入市政公共设施建设。

切实减轻农民负担。落实减轻农民负担行政首长负责制 和部门责任制。巩固农村税费改革成果，全面免征农业税。认真查处侵犯农民合法权益的违法行为，深入开展涉农不合理收费专项治理，防止农民负担反弹。继续落实扶贫政策措施，实施“农村安居工程”和“千村扶贫工程”，集中力量扶持集体经济收入3万元以下的贫困村，加快贫困地区脱贫奔康步伐。

三、推进市场流通，搞活商贸经济

加大外贸出口力度。调整优化出口产品结构，大力培育有自主知识产权和自主品牌的产品出口，提高出口商品科技含量和附加值。巩固港澳、欧美日、东盟等现有市场，大力开拓非洲、南美、俄罗斯等新市场。落实加工贸易出口考核任务和奖罚措施，积极发展加工贸易。继续实行出口退税共同负担机制，加快退税进度，推动外贸出口稳步增长。加强与海关、检验检疫、税务、外汇管理等部门的协调和沟通，营造良好的“大通关”环境。

加大商贸流通力度。采取“政府搭台，企业唱戏”的方式，组织企业参加省举办的各类经贸洽谈会、展销会。继续办好中华阳美（国际）玉器节、普宁（国际）衬衣节暨纺织服装交易会、中国（普宁）中药交易大会，筹办揭西生态旅游文化节，积极做好“中国玉都”、“中国纺织服装城”申报工作，促进会展经济的发展。建设市区金属材料、粮食市场和大型购物广场，搞活粤东鞋材城、水果批发等市场，做大做强普宁中药材、纺织服装、布料等专业市场。大力发展旅游业，加大旅游产品宣传推介力度，加快旅游资源的保护、开发建设。努力拓宽消费领域，积极发展物业管理、社区服务、中介服务等行业，促进连锁经营、现代物流、网上购物等新型业态的成长，培育新的消费热点。

加大对外协作力度。认真实施与华南理工大学教育、科技合作协议，尽快发挥效益。继续推进闽粤赣十三市区域经济协作，加强同“泛珠三角”、“大珠三角”区域的联系和交流。积极创造条件，引进有利于我市协调发展的产业，拓展有利于我市发展的领域。

四、推进县域发展，夯实地方经济

大力发展特色经济。充分依托县域资源优势和产业比较优势，因地制宜，打造“四大产业带”。发挥揭东、榕城、东山和试验区制造业基础好、区位环境好的优势，大力发展五金不锈钢、化工、塑料、鞋类、电子电器、模具、轮胎等制造业，打造沿江经济产业带；发挥普宁工业商贸基础较好的优势，大力发展纺织服装、医药、食品和现代物流业，打造中部经济产业带；发挥惠来82公里海岸线的独特优势，大力发展能源、化工、旅游、海运、海水养殖和加工业，打造沿海经济产业带；发挥揭西生态环境好的优势，大力发展生态农业、生态工业和生态旅游业，打造西部经济产业带。普侨区和大南山侨区要作为工业园区基地，加大开发力度。

大力发展民营经济。认真贯彻落实国家、省、市加快民营经济发展的优惠措施，放宽市场准入，建立贷款担保等社会中介机构，逐步完善投资服务和信用评价体系，帮助民营企业解决融资难等问题，继续开展表彰民营企业纳税光荣户活动。培育一批专精特的中小企业，形成集群产业和块状经济。

大力发展劳务经济。依托各类职业培训机构，加强职业技能培训，提高劳动者的素质和技能。抓好10个劳动力市场、100个基层劳动保障平台软硬件设施建设，完善公共就业服务体系。加强农民外出务工的信息引导、管理和服务，争取培训农村富余劳动力5万人，转移输出4.2万人。

五、推进重点建设，增强发展后劲

加强能源建设。争取惠来电厂尽快通过国家发改委核准。加快惠来石碑山风电场、500千伏榕江输变电工程和紫峰、铁山、华湖等6个220千伏输变电工程建设，确保500千伏长沙至揭阳、220千伏棉湖至紫峰线路工程和棉湖220千伏输变电扩建工程于年底前投产。加快东方、磐东等15个新建、续建110千伏输变电工程和市区配网建设。继续开展农村电网建设与改造，进一步完善电网结构。力争核电项目落户惠来。

加强交通建设。争取潮汕民用机场今年通过国家发改委核准，明年初开工。积极协助做好汕揭、潮揭高速公路建设。加快国道206线揭丰段、省道236线池揭段改造和南河大桥扩建工程建设步伐。认真协助做好厦深铁路规划和筹建工作。加快县通镇、镇通行政村公路改造，确保按期完成。

加强水利建设。全力推进市区防洪工程建设，做好榕江大围的打捆上报工作，争取列入国家投资计划。力争惠来赤岑水闸尽快立项投建，加快惠来石榴潭水库、揭东磐岭围、普宁流沙联围新河堤加固和揭西县城防洪工程、金凤联围等项目建设进度，提高城乡水利防灾减灾能力。

六、推进市区建设，构建区域核心

培育经济优势。按照把市区建成100万人口以上大城市的要求，完善市区总体规划、16项专项规划和小区规划，调整优化市区发展布局，提升并形成中部（榕城区）商贸文化教育、东部（试验区）高新技术产业、北部（东山区）金融信息商贸、西部（磐东）和南部（仙桥、梅云）传统工业五大功能片区。做大做强市区工业，构建粤东商品集散中心，培育发展高新技术、信息和金融产业，壮大市区经济实力，发挥龙头带动作用。

培育体制优势。树立经营城市理念，整合城市资源。严格控制、整合利用58平方公里内未开发土地和两河四岸建设线之外纵深150米土地。对城市公交线路、户外广告、地下管道、地下水及河沙等资源实行有偿使用，走以城建城、以城养城、滚动发展的路子。推进公用事业改革，建立资源配置机制，通过整合人才、文化、能源、土地、金融等资源，使城市核心区迅速发展起来。

培育环境优势。修改完善《揭阳市城市管理行政执法暂行规定》，分类清理250万平方米违章建筑。加强市区房地产市场管理，引导建设时尚高档商住区。推动城市管理重心下移，努力形成齐抓共管工作格局。深入开展城市综合治理，逐步用公共汽车取代营运三轮车、摩托车，努力改变城市“脏乱差”现象，创造整洁、有序、祥和的社会环境。

培育人文优势。广泛宣传《揭阳市市民文明公约》、《揭阳市公民道德规范》，不断提高市民文化道德素质，引导市民养成科学、健康、文明的生活方式。加大历史文化名城保护力度，围绕双峰寺、城隍庙、学宫三个具有代表性的传统文化景观，开辟文化广场，构建市区文化中心。修缮原有公园，新建临江、望江等公园，逐步完善文化、教育和旅游、休闲娱乐设施建设，营造有利于市民健康成长、安心生产、舒心生活的人文环境。

培育市政优势。力争4年内筹集30亿元以上资金投入市政建设，使城市功能上档次、上水平。做好“水”字文章，构建两河四岸特色风景带。强力推进旧城改造，建好管好市区四个主要出入口，规划、建设好城市灯光亮化工程。加快莲花大道南段和临江北路、黄岐山大道等4条样板路建设，抓好天福东路等“断头路”、“烂尾路”和破损路面的修建，完善汽车站、公共汽车上落站、十字路口红绿灯、电话亭、公共厕所、垃圾箱、消防栓等市政公共设施。推进码头规划建设，尽快形成水道、公路、铁路、机场配套完善的水陆空立体交通网络。

七、推进各项事业，统筹社会发展

优先发展教育事业。成立市振兴教育领导小组。落实基础教育“以县为主”的管理体制，巩固提高“普九”成果。大力发展高中阶段教育，加快幼儿教育和特殊教育发展步伐，继续发展中等职业教育和高等教育。多渠道筹措资金，力争今后3年全市基础教育投入16.8亿元，今年投入6.2亿元。积极稳妥调整中小学布局，新建、扩建一批高中、初中和小学，加强薄弱学校改造。加快揭阳一中新校区建设进度，确保明年春季开学投入使用。推进市技校新校区建设，争取首期工程今年秋季竣工。做好市卫校新校区建设前期工作。办好揭阳学院和潮汕学院。深化办学体制改革，鼓励和规范社会力量办学，积极探索国有民办、公办民助、民办公助等办学形式，形成公办和民办教育共同发展、相互促进的新格局。积极推行校长聘任制和教师岗位责任制，加大教师培养、引进和使用力度，着力解决中小学师资力量不足和结构不合理等问题，全面推进基础教育课程改革，提高教育教学质量。构建学校、家庭、社会思想道德教育网络，全面提高师生思想道德水平。

大力繁荣社会文化。不断深化文化体制改革，整合文化资源，打造文化产业品牌。努力繁荣文艺创作，保护和发掘地方特色优秀历史文化。大力发展广播电视、新闻出版、文史档案等事业。加强文化市场管理，进一步规范文化市场秩序。推进“文化工程”，尽快动工建设市广电中心、图书馆，筹建市体育馆、档案馆。加快县、镇公共文化设施建设，争取全市乡镇文化站基本达标。

积极发展科技事业。加大科技投入，加快科技创新步伐，积极培植和申报国家、省级高新技术企业及省级民营科技企业，力争新增2个以上工程技术研究开发中心、10家以上省级民营科技企业。加强科技示范工作，加速科技成果转化。做好科普知识宣传工作，依法保护知识产权。

努力抓好人才工程。加快人才政策法规建设，建立完善人才评价、选拔使用、分配激励、保障和流动五大机制，营造有利于各类优秀人才创业、工作、生活的社会环境。

促进其他各项事业发展。广泛开展全民健身活动，大力发展竞技体育，办好市第四届运动会。认真开展经济普查。抓紧编制好“十一五”规划。加强国防安全教育，做好双拥工作。发展老龄、妇幼和残疾人事业。全面贯彻民族宗教政策，依法管理宗教事务。进一步做好外事、侨务、民政、人防、方志、气象、地震等工作。

八、推进各项改革，建立良好机制

深化财政管理体制改革。在确保各县（市、区）既得利益的前提下，建立健全市与县（市、区）财政管理体制。按照事权与财权相统一的原则，调整理顺县级与镇（乡、街道）财政管理体制，调动基层协调税收和社保费征管的积极性。建立财税收入增长监测分析新机制，强化税收征管，切实做到应收尽收，确保财税收入与经济增长良性互动、协调发展。严格规范各种规费的收取行为，坚决禁止部门不作为和违规现象。健全财政、审计监督机制，强化支出管理，提高财政资金使用的规范性、有效性和安全性。

深化国有企业改革。以产权制度改革为核心，完善国有资产管理体制和监管方式。鼓励民资和外资参与国有企业改革改组改造，加快完善现代企业制度。今年要作为国有企业改革攻坚年，加大改革力度，采用一厂一策、逐个解决的办法，积极筹措资金，妥善做好下岗职工解除劳动关系工作。争取通过3年努力，完成全市国有企业改革任务。

深化投资体制和市场体系改革。落实企业投资自主权，推行企业投资项目核准制和登记备案制。实行投资项目绩效综合考评，健全投资决策责任制。大力发展资本、人才、土地、劳动力等要素市场，规范发展产权交易市场。继续深化粮食流通体制改革。

深化农村各项改革。做好第二轮土地承包后续管理工作，引导和规范农户承包土地经营权的有序流转，加强农村集体征地补偿款分配和使用的监督管理。全面实行村账镇管制度，做好集体资产清产核资、资产评估和审计监督工作，积极稳妥化解镇村不良债务。继续推行“两公开一监督”，完善村务公开和民主管理制度。依法推进撤并村镇工作。认真做好村委会换届选举工作。 深化地方金融体制改革。加强与金融部门的联系和协调，推进政银企合作，积极开展质押业务。打击逃废债务行为，稳定地方金融秩序。加大地方金融资产、财政资金追收处置力度，积极防范、化解地方金融和财政风险。认真抓好农村信用社改革试点工作。

九、推进各项管理，营造和谐社会

高度关注民生民计。建立健全再就业工作目标责任制，加大就业开发力度，增加就业岗位2万个，实现下岗失业人员再就业0.5万人。加强社保扩面征缴和基金管理，完善市级统筹，确保社会保险待遇按时足额发放。健全公共卫生应急体系，加强农村卫生工作，强化疾病控制和卫生监督，完善新型农村合作医疗制度，争取今年全市覆盖率达到50%。健全城乡最低生活保障制度，做好帮扶弱势群体工作，着力解决群众子女入学难、看病难、打官司难、住房难等问题。扎实推进农村饮水工程建设，缓解群众饮水难状况。做好救灾救济工作。大力提倡团结互助、友爱向善、扶贫济困的良好风尚，促进社会全面进步、人的全面发展和全市人民和谐相处。

加强生态环境保护。实行环保目标任期责任考核，把环保责任制和追究制落实到各县（市、区）、镇（乡、街道）和村（居）。大力实施“环保工程”，加快市区垃圾处理场、污水处理厂和普宁流沙、揭西河婆污水处理厂等环保工程建设步伐，抓紧完善治污配套设施。加大执法力度，坚决取缔严重污染的企业，禁止沿江居民向江河乱倒垃圾，强化“三江”环保综合整治。重视城镇社区特别是主干道路的植绿、护绿工作，抓好榕江南北河两旁生态公益林建设，加强土地、江河、海洋等资源管理，提高资源综合利用水平。坚持计生“三为主”方针，健全计生工作“七项机制”，确保完成计生工作任务。继续完善殡葬基础建设，巩固殡改成果。

严厉整治赌博活动。坚决从源头上禁赌，保持高压态势，重典治赌，坚决拆保护伞、铲庄家、端赌窝，拆散赌博网络，斩断海内外伸进揭阳的黑手，在全市范围严惩赌博犯罪活动。加强舆论宣传，形成全市上下齐动打击赌博犯罪活动的良好局面，还揭阳人民一个清静干净的社会氛围。

努力维护社会稳定。依法严厉打击各种刑事犯罪，特别是严重暴力性犯罪、黑恶势力犯罪和“两抢两盗”等多发性犯罪活动，继续实施“禁毒03工程”，建立健全群防群治网络，推进社会治安防控体系建设。做好人民群众来信来访工作，及时妥善处置群体性事件。全面排查社会矛盾，落实基层维稳责任，把矛盾解决在萌芽状态，解决在基层。严厉打击制假售假、走私贩私、偷税逃税等违法犯罪活动，进一步规范市场秩序。深化安全生产专项整治，防止重特大安全事故的发生，确保人民生命财产安全，保持社会稳定。

十、推进民主进程，建设法治政府

自觉接受监督。自觉接受人大及其常委会的监督，认真落实人大及其常委会的决议、决定，及时向人大及其常委会报告工作，切实办好人大代表议案、建议。积极支持人民政协履行政治协商、民主监督、参政议政职能，重视政协委员提案办理工作。认真听取各民主党派、工商联、无党派人士和工会、共青团、妇联等群团组织的意见建议。自觉接受社会舆论监督、审计监督和行政执法监督，进一步推进政务公开，增强工作透明度。

推进依法行政。加大“四五”普法力度，营造全社会崇尚法律的良好氛围。认真贯彻落实国务院《全面推进依法行政实施纲要》，严格按照法定权限和程序行使权力，规范行政行为，做到执法有保障、有权必有责、用权受监督、违法受追究、侵权须赔偿。完善专家咨询、社会公布和听证会等制度，探索推行政府法律顾问制度，促进政府决策科学化、民主化、法制化。

加强自身建设。改进政府管理经济和社会的方式方法，提高政府领导经济、管理社会的水平。改进工作作风，精简会议文件，做到察实情、办实事、求实效。建立健全各种预警和应急机制，提高保障公共安全和处置突发事件的能力。完善政绩考评办法，健全绩效评价体系。落实廉政建设责任制，认真预防和严厉惩治腐败犯罪，坚决纠正损害群众利益的不正之风。开展“理想、责任、能力、形象”教育活动，提高队伍素质，建设一支政治坚定、业务精通、清正廉洁、作风优良的公务员队伍，努力建设务实高效、法治民主、人民满意的政府。

各位代表，让我们在市委坚强有力的领导下，同心同德，开拓创新，团结奋进，为实现我市经济社会发展新跨越而努力奋斗！
